# Editorial: The impact of sedentary behavior and virtual lifestyle on physical and mental wellbeing: social distancing from healthy living

**DOI:** 10.3389/fpubh.2023.1265814

**Published:** 2023-08-03

**Authors:** Fahad Hanna, Emily You, Mohamed El-Sherif

**Affiliations:** ^1^Public Health Program, Torrens University Australia, Melbourne, VIC, Australia; ^2^Higher Education College, Chisholm Institute, Dandenong, VIC, Australia; ^3^Academic Unit for Psychiatry of Old Age, Department of Psychiatry, The University of Melbourne, Melbourne, VIC, Australia; ^4^Department of Bariatric Surgery, Hamad Medical Centre, Doha, Qatar

**Keywords:** sedentary lifestyle, physical activity, COVID-19, physical health, mental health

## Introduction

In recent decades, technological advancements have transformed the way we live and work, offering convenience and comfort. However, this comfort comes at a cost to our health. These advancements result in increasingly prevalent sedentary lifestyle and inactivity which negatively impacts our wellbeing. The adverse effects of prolonged sitting and physical inactivity are not only limited to the present generation; they also pose a serious threat to our future, as they interfer in human natural growth and development (1). Therefore, it is vital to assess the profound impact of sedentary behavior on human health to better inform policies, and health interventions or programs to combat this growing crisis.

While the human body has basic need for movement and physical activity (2), our modern lifestyle has significantly reduced our daily activity levels, resulting in a multitude of health problems (3). Presta et al. in this Research Topic described how “engaging in regular physical activity (by playing sports and being as active as possible during the daily routine)” is evidently protective against “NCDs and NCDs-related risk factors, namely overweight and obesity, and hypertension.” The combination of obesity and sedentary behavior has also been negatively associated with longevity and life expectancy (4). Being active in general has also been linked to quality of life. A Chinese study published in this Research Topic found that the combination of good level of physical activity and reduced sedentary behavior has a positive impact on the quality of life among children and adolescents (Shi et al.).

Consequences of prolonged sedentary behavior relevant to occupational settings have also been assessed extensively. Musculoskeletal disorders, including back pain, neck pain, and joint problems, are widespread among individuals with sedentary jobs. Prolonged occupational sitting leads to excessive strain on the spine and muscles, ultimately causing postural imbalances and chronic pain (5). The above study added that the lack of physical activity and increased sedentary time may increase the risk of developing mental health issues. A study in this Research Topic found that screen-based sedentary behavior was associated with anxiety symptoms among college students (Huang et al.). Other studies showed clear links between sedentary behavior and increased levels of anxiety and depression, as well as decreased cognitive function (6). The plausible mechanism is that lack of physical activity limits the release of endorphins, serotonin, and other mood-enhancing chemicals, resulting in a higher risk of mental health disorders.

When the COVID-19 pandemic hit, governments around the world moved fast to declare emergencies and implemented policies that restricted movement across all ages, occupations and educational institutes. We started to work and study remotely and the “social distancing” that was designed to protect us from the virus ultimately led to “social distancing” from healthy living. Two studies in this Research Topic addressed the impact of COVID-19 pandemic measures and fear of transmission and spread, on the reduced level of physical activity and increased level of sedentary behavior and subsequent health outcomes. Al-Hindawi et al. found that 2 in 3 medical and nursing students reported a decrease in physical activity and increase in sedentary behavior during the pandemic. Another study conducted by Shpakou et al. compared countries with “different approaches of anti-pandemic measures” and reported decline in levels of physical activity among university students and students athletes. The above study added that lower levels of physical activity in one of the countries were associated with lower life satisfaction and lower levels of ability to cope stress (Shpakou et al.).

Pandemic or no pandemic, the countless health threats associated with unhealthy lifestyle and behavior have undoubtedly gotten the attention of researchers in recent times. A community-based study in this Research Topic conducted by Zhang et al. addressed the impact of “unhealthy lifestyle” prior to the pandemic. This study reported that participants with unhealthy lifestyle scores, including physical inactivity and sedentary behavior, were more likely to report depressive symptoms (Zhang et al.). The above unhealthy scores got worse with additional unhealthy behaviors such as smoking, sleep deprivation and excessive alcohol consumption, indicating accumulated risk, and subsequently, further decline of health and wellbeing.

## Conclusion

There is no doubt that sedentary lifestyle poses a serious threat to human health and wellbeing, which is aggravated by the pandemic. Considering the pandemic and other forms of large-scale disasters that warrant lockdowns and long-term restriction on movement, public health planners and policy-makers should develop and implement appropriate strategies to reduce sedentary behaviors in the community, particularly those at risk. However, addressing the reality of sedentary lifestyle entails a multifaceted approach and partnership with individuals, communities, and policymakers. Workplace interventions and programs should aim at reducing sedentary time and promoting a culture of healthy lifestyle and wellness. Moreover, public health campaigns and interventions should focus on raising awareness of prolonged sitting and inactivity and encourage undertaking physical activity compromising by adhering to public health orders of not spreading the infectious disease during pandemics.

## Author contributions

FH: Conceptualization, Investigation, Resources, Supervision, Visualization, Writing—original draft, Writing—review and editing. EY: Supervision, Validation, Writing—review and editing. ME-S: Conceptualization, Project administration, Visualization, Writing—review and editing.
